# A peer-and self-group competitive behavior-based socio-inspired approach for household electricity conservation

**DOI:** 10.1038/s41598-024-56926-1

**Published:** 2024-07-27

**Authors:** Gaikwad Sachin Ramnath, R. Harikrishnan, S. M. Muyeen, Amit Kukker, S. D. Pohekar, Ketan Kotecha

**Affiliations:** 1 Symbiosis Institute of Technology (SIT), Pune Campus, Symbiosis International (Deemed) University, Pune, India; 2 Department of Electrical Engineering, Qatar University, 2713 Doha, Qatar; 3 Department of Computer Science Engineering, Chandigarh University, Chandigarh, India; 4 Symbiosis International (Deemed) University, Pune, India; 5 Symbiosis Centre for Applied Artificial Intelligence, Symbiosis Institute of Technology, Pune Campus, Symbiosis International (Deemed) University, Pune, India

**Keywords:** Socio-technical behavior, Household electricity conservation, Peer-and self-group learning, Genetic algorithm, Knowledge-based decision system, Electrical and electronic engineering, Power distribution

## Abstract

This paper proposes a knowledge-based decision-making system for energy bill assessment and competitive energy consumption analysis for energy savings. As humans have a tendency toward comparison between peers and self-groups, the same concept of competitive behavior is utilized to design knowledge-based decision-making systems. A total of 225 house monthly energy consumption datasets are collected for Maharashtra state, along with a questionnaire-based survey that includes socio-demographic information, household appliances, family size, and some other parameters. After data collection, the pre-processing technique is applied for data normalization, and correlation technique-based key features are extracted. These features are used to classify different house categories based on consumption. A knowledge-based system is designed based on historical datasets for future energy consumption prediction and comparison with actual usage. These comparative studies provide a path for knowledgebase system design to generate monthly energy utilization reports for significant behavior changes for energy savings. Further, Linear Programming and Genetic Algorithms are used to optimize energy consumption for different household categories based on socio-demographic constraints. This will also benefit the consumers with an electricity bill evaluation range (i.e., normal, high, or very high) and find the energy conservation potential (kWh) as well as a cost-saving solution to solve real-world complex electricity conservation problem.

## Introduction

The recent global rise in energy demand, adversely affecting environmental development is discussed in^1,2^. According to the Paris Climate Conference-21 summit, a legally binding target has been set for all economic sectors to limit the effects of climate change, by controlling the global mean temperature to below 2 °C^3^. Furthermore, in 2011, the International Energy Agency mentioned that the domestic sector worldwide had a more significant energy-saving potential of about 0.48*106Ktoe per year. Additionally, the global domestic energy consumption share is approximately 25% which is the one of the largest overall consumption, contributes to 17% of CO_2_ emissions in overall CO_2_ emissions, as reported by the IEA in 2016^1,3^. According to the IEA 2023 report, the cost of energy for the advanced economies to residential consumers will be nearly 20% that will be reduced by 2030 due to reduced fossil fuel consumption and increased energy efficiency. The countries must provide subsidies for renewable energy generation to overcome energy costs and reduce CO_2_ emissions for emerging markets. The Organization for Economic Co-operation and Development countries significantly contributes to national carbon emissions, with the United Kingdom contributing 25%^3,4^. Due to the effective implementation of the EE-based Domestic Appliances policy during the past 40 years, it is observed that household appliances consume only 2% of electricity^1,3^. During field visits for data collection, it is observed that household consumers compare their Energy Bill (EB) (abbreviated as EB, shown in Table 1) with their neighbors and/or similar households to know whether the received EB is normal or high. This is a human tendency to compare EB and find appeal in them. This comparison may not always lead to informed decision-making because manual meter reading and billing processes may generate error bills. Meanwhile, in the manual meter reading and billing systems, the two significant sources of error are (i) human error and (ii) machine error^5,6^. These types of error may also generate the average bill to end consumers.

**Table 1:** Abbreviations used.

Symbol	Description	Symbol	Description
EB	Energy bill	MC	Monthly energy consumption
EBR	Energy bill report	MH	Medium houses
EC	Energy conservation	P	Peer-comparator
EE	Energy efficiency	S	Self-comparator
FE	Feature engineering	SA	Social algorithm
GA	Genetic algorithm	SH	Small houses
HEC	Household electricity consumption	VLH	Very large houses
LH	Large houses	Qs (Sum)	Sum of important attributes of QS
KDSEO	Knowledge-based decision-making System for energy bill evaluation and optimization	QS	Questionary survey
SHOEC	Socio-technical household optimized electricity conservation	kWh	Kilo watt hour
SCB	Socio-technical competitive behavior	Avg_kWh	Average energy consumption
HEC	Household electricity consumption		

Furthermore, the utility company has its energy bill verification system before the generation of the energy bill. This verification system is generalized and threshold-based, applicable to all consumers. Further, the energy bill verification process of utility companies varies and mainly depends on the number of consumers, areas/locations, availability of human resources, and adopted technology. They will verify energy bills only if the monthly energy consumption of the consumer is four times the average monthly consumption^5^. To impose a rule or limit on all consumers may not be practical and appropriate, as this can lead to inadvertently high or erroneous energy bills for consumers. Thus, only a generalized threshold-based approach may be effective and equitable for some consumers^7^. Due to this, consumers and even utilities would be unaware of the actual energy consumption. Household Electricity Consumption (HEC) is a technical term with a dynamic, complex, uncertain nature and it depends on many factors. The HEC needs a problem-specific, innovative approach to understand the consumption pattern and critical factors, which helps work on areas like energy efficiency, energy conservation, energy management, load management, planning and policy-making, and economic analysis^3,8^. These factors will guide technology development, cost savings, consumer behavior change, social and environmental impact research, and solutions^9,10^. The present work aims to solve this problem by proposing a Knowledge-based Decision-making System for Energy bill Evaluation and Optimization (KDSEO) for competitive analysis of household energy savings. The motivation is to use a nature-inspired algorithm, that is cultural or social optimization. The human behavior tendency of comparison between peer and self-groups based on the proposed socio-technical Competitive Behavior (SCB) approach is utilized to design a knowledge-based decision-making system. Thus, KDSEO generates an energy bill evaluation report at the consumer end, that will allow them to compare their bills (that is high, low), check for errors, and finally make informed decisions^11^.

The Genetic Algorithm (GA) approach is used to calculate optimal energy consumption for different house sizes using objective functions, which are based on house size, number of members in the house, number of electrical appliances used in the house and other parameters with different energy consumption constraints. The genetic algorithm is an optimization algorithm based on natural selection and genetic principles. It is a heuristic search algorithm used to find the optimal solution to a problem by mimicking the process of natural evolution. A genetic algorithm begins with an initial population of candidate solutions, represented as chromosomes in a genetic metaphor. Each chromosome is a vector of values representing a possible solution to the problem. The genetic algorithm then applies genetic operators, such as selection, crossover, and mutation, to these chromosomes to generate new generations of candidate solutions. Here, a solution is the optimal energy consumption value for different house categories^12,13^.

The organization of the paper includes various Section. The second Section is the Background. The third Section is the Methodology, which discusses the implementation of a socio-technical household electricity conservation algorithm based on the socio-inspired optimization. The fourth Section is the Results, including KDSEO, Linear Programming, and GA. The fifth Section is the Discussion and Conclusion, which discusses the findings and summarize the work.

## Background

Energy Efficiency (EE) and Energy Conservation (EC) codes, standards, policies, and regulations are in the present global growing economic development, amplifying demand for energy and climate change circumstances. Moreover, today's needs in power sectors are to achieve effective building energy management from a cost-effective perspective. The buildings are mainly classified into four categories: commercial, residential, educational, research, and other types of buildings. Residential building consumption contributes the largest share of energy^14^. A recent study shows that buildings consume more than 50% of global energy, including a share of 33% thermal load efficiency, and carbon emission is around 30% of the world's carbon emission. The need for electrical energy has been rising significantly. The result shows the increasing citizenry's standards of living. A larger population, the effects of urbanization, socio-economic development, large-scale machinery, and electronic market trading are additional factors^15^. However, with an increase of about 25%, global domestic energy consumption is now the second most significant component of the total consumption. Meanwhile, according to the International Energy Agency (IEA), 2016^2^, CO_2_ emissions are overall increasing by 17%^1,16^. Energy waste is a growing concern, as it negatively affects the environment. As a result, policymakers or decision-makers should pay close attention to the need for energy efficiency and energy conservation at the building level^17^. Despite this, more research is needed in the residential sector compared to other industries or buildings^18^. The main challenge is that energy consumption is dynamic; consumption depends on multiple direct and indirect factors, lack of data availability, less accuracy of the prediction model, ideal response of the proposed model, and installation of inadequate smart meters. Thus, due to the massive scope and potential, more research is needed in residential buildings^16^. Compared to the developed countries in developing countries, household energy consumption based on research is less^14^.

Domestic electricity consumption is second in total power consumption in India, 24%^18^. Despite this, there is increasing appliance ownership and electricity consumption due to increasing of the electrification of households, improving the income level, and technological development in the Indian power sector. Furthermore, research has been done in the use of domestic electricity in India. Integrating Engineering, Economics, Anthropology and Architecture are needed to understand occupants' consumption behavior^19^. In households, the end-users have different types of loads. The household energy consumption analysis can be done based on loads like whole-building or household electricity, heating and cooling, only heating, only cooling, only lighting, and all others^14,20^. Significant work was found on household electricity consumption-based load and cooling load modeling. More research is needed on the lighting load type compared to other load types. Lighting contributes to around 20% of the total electricity consumption of the world. Moreover, around one-third of the cooling load can be conserved by reducing the lighting load, using more artificial light and solar heaters^16^. The literature supports the need for more research on real-world data on the residential-building sector, specifically in developing countries^14^. Based on the proposed research gap, the research question is "What are the influencing primary factors and the challenges faced for improving the energy efficiency of household electricity consumption studies in the context of developing countries?

The data-driven approach consists of four primary steps: Data collection, Preprocessing, Model training, Model testing, and Validation^14,16^. The literature discussed three data types: Real, Simulated, and Public benchmark data. Mostly real data collection uses conventional energy meters, smart meters, and sensor-based help for real-time data collection. Smart meters and sensor-based data collection methods need additional installation costs and privacy issues. The electricity consumption prediction of residential buildings is costly, time-consuming, and involves privacy issues in data collection^14^. However, for a better understanding of the ground reality of household electricity consumption, there is a need to work more on ground-level studies like a Questionnaire-based survey. This helps to collect the real datasets and focus on solving the applied and action type of research problem statement^2^. Despite this, literature discussed standard methods for data collection applied in household electricity consumption studies, namely personal interviews, telephone surveys, energy meter readings, household electricity consumption pattern monitoring, individual appliance consumption using sub-meter, questionnaires-based surveys, energy audits, National or Regional level household surveys, and monthly bills of the ESP company^2,21^.

Many papers applied questionnaire survey data collection techniques in data-driven energy models. The questionnaire survey design, data quality, and its sufficiency are essential to achieve the quality outcomes from the study^1,21,22^. Additionally, a statistical approach is critical in real data collection-based studies. Many works still need to clearly define why they selected a particular sampling method, how they have fixed the sample size, and what steps good practices were applied while designing the sample survey^1,22,23^. This paper addresses this gap by implementing real data collection using a Questionnaire-based sample survey method. The subsection discussed the sample size, sampling strategy, and questionnaire design with good practices and discussed the factors affecting the response rate^1,24,25^. On the other hand, the large-scale and complex research study needs to apply the Multistage survey technique. Alternatively, it will help to increase the response rate and quality of the collected data^26,27^. A systematic design of the questionnaire has observed positive responses from the participants with quality data collection. According to the literature, the response rate was raised for those questionnaire surveys, which included short, precise, and meaningful questions. As far as possible, personal questions are avoided. However, there is a lower response rate for Sensitive questions-based surveys such as smoking habits or alcohol and drug habits. Moreover, network sampling is used to contact the respondent for more responses^27,28^. From the literature support, we come to the research gap of many researchers who need help to spot systematic data-collection methodologies. Based on the proposed research gap, the question is, "How do you carry out systematic data collection and preprocessing?

The occupant's behavioral intervention directly impacts their decision-making to buy and use the appliances. Income level, climate, culture, and social values can also affect human behavior and its impact on electricity consumption. In^29^. A case study considered 500 households around Delhi and proposed the effects of comparing the electricity bills of the neighborhood, which resulted in 8% reductions in the electricity bills of the occupants. It showed the importance of human intervention and the effect of behavioral economics on the reduction of the electricity bill. It proves appreciably helpful for the Indian context. The interdisciplinary modeling approach helps to find the root causes of changing electricity demand. The electricity consumption research works less with a psychological approach to know human behavior and its effects on various activities and decision-making on electricity consumption^30^. The sociology approach helps to understand the reasons for variations in the consumption behavior of society, culture, technology, and history^31^. The results of the modeling approach should be different from the 'ideal,' reflecting the actual on-ground reality of the electricity consumption pattern. India has not conducted intermittent Residential Energy Consumption Surveys (RECS) like other countries. Concerning NITI Aayog, the 2012 report shows the various possible energy circumstances until 2047, representing the state-of-art housing electricity consumption^19^. We come to the research gap of limited research on interdisciplinary approach-based household electricity consumption studies from the literature support. Based on the proposed research gap, the question is, "Why is it necessary to constitute an appropriate multidisciplinary research approach?

Table 2 summarizes the literature survey, including system input, EC (Energy Conservation), EB (Energy Bill), and outputs through applied methodology and results.

**Table 2:** Summary of literature survey on HEC and socio-inspired approach.

References	Topic	Inputs	Outputs: Methodology/Results
^6^	Both Energy conservation and Energy Bill Analysis	Arduino and GSM to get the unit consumption of each house	Addressed manual energy billing mechanism issues and power shutdown
			An automatic EB calculation system has been proposed
			This study is lacking in providing the system on the consumer side to evaluate monthly EB and its optimization approach
^31^	Both Energy Conservation and Energy Bill Analysis	PV source	Multi-agent reinforcement learning
		Energy bill	PV generation and load scheduling to reduce EB
^32^	Both Energy conservation and Energy Bill Analysis	Renewable sources	The load classical optimization method to develop the Greedy Randomized Adaptive Search Procedure (GRASP) algorithm
		Energy bill	Adjusting the demand peaks and low computational time
			Linear modeling approach was used
^33^	Both Energy conservation and Energy Bill Analysis	Energy audit	Energy audit-based approach is used to implement DSM
		Survey	DSM aims to obtain minimal cost planning or integrated resource planning
			DSM was validated using BPSO with constraints of peak-to-average ratio and cost reduction
			MATLAB simulation tool is used
			Due to large rating devices used in the industry, more profit was obtained for load DSM
^34^	Both Energy conservation and Energy Bill Analysis	In-house display information	To optimize the HEC using a feedback mechanism within the house display
			The main challenge is if the feedback is noisy
^35^	Both Energy conservation and Energy Bill Analysis	Energy-storable and non-storable appliances are considered	Addressed the energy wastage issue
			Integrated solar PV sources for EB optimization
^36^	Both Energy conservation and Energy Bill Analysis	SCADA	Proposed the appliance controlling algorithm for household EC and EB optimization based on dynamic energy pricing
		Solar PV panels, Battery banks, hybrid vehicles	The study says that SCADA can balance the minimization of EB of users with adjusting peak load demand for utility and limiting carbon emissions
^37^	Energy conservation	QS for households	Variables used: construction features, energy usage, and users’ income level attributes
			The old AC replacement has a 32% annual energy-saving potential
			Around 14% of houses followed EE programs and adjusted the AC temperature above 26 °C
			EE and EC programs should be revised based on requirements
^38^	Energy conservation	QS for households	Applied various techniques: descriptive statistics, machine learning, and regression analysis
			The AC usage in households is mainly increased due to AC usage at the workplace, increased income, and maintained social status
^39^	Energy conservation	Real-time energy and resource management pricing signals	Discussed the DSM effectively utilizing demand-side resources and distribution infrastructure
			Simulated work aims to maximize social welfare by developing an optimization model
			The optimization model has been developed to decentralize work, further used to achieve social welfare
^9^	Cost optimization	Review	Cost optimization using the Particle Swarm Optimization algorithm is applied
		Load data	The application of the proposed work can be helpful for load forecasting, scheduling, and management
^40^	Energy Bill Analysis	Energy consumption data	DSM framework, techniques, optimization models, and methods are discussed
			Highlighted that the heuristic, stochastic optimization techniques and game theory are essential to deal with DSM for solving complex and dynamic energy load management problems
^41^	Energy Bill Analysis	Solar PV source	Case study based on 2BHK residential flat
			Renewable distributed generation can be integrated into the conventional grid system to reduce the EB
			Defined the objective cost function for EB optimization through a simulated linear programming approach
^42^	Load Profile classification	Load profile data	Worked on a systematic framework for load profile classification like human-readable and machine-readable
			The data mining techniques like classification and regression trees are used to extract the features in the frequency domain
			A hierarchical classification tree is a more effective and systematic method for load profile classification
^43^	Cost optimization in microgrid	Real data of non-dispatchable resources and non-responsive loads	Aims for optimum operation strategy of microgrid by cost optimization and demand response regulation
			Implemented a multi-period imperialist competition algorithm with an expert heuristic approach
			ANN and the Markov-chain method are applied to predict non-dispatchable power generation and load demand under uncertain conditions
^44^	Socio-inspired optimization	Group of families	This work highlighted that the social evolution and learning way is faster to influence behavioral change than the genetic evolution and learning method
			The work has provided ground to explore the metaheuristic optimization approach to solve real-world, specific, and complex problems
^45^	Socio-inspired optimization	Group of families	Discussed Multi-Cohort Intelligence (CI) metaheuristic algorithm using intra-group and inter-group learning mechanisms
			The prominent features and limitations of the Multi-CI algorithm have been discussed
			The Multi-CI algorithm can be further used to solve real-world and constrained test problems
			MATLAB tool is used for simulation
^46^	Socio-inspired optimization	Group of families	Proposed a socio-inspired optimization-based ideology algorithm
			Proposed work applied self-interested, competitive behavior evolution and learning approaches in political applications to improve their rank
			This work improved the optimization performance regarding objective function values and computational time
			MATLAB tool is used for simulation

As behavioral economics affects the reduction of electricity bills, human intervention is essential. It proves appreciably helpful for the Indian context^1–4,29^. The interdisciplinary modeling approach helps to find the root causes of changing demand of electricity. The electricity consumption research works less with a psychological system regarding human behavior and its effects on various activities and decision-making on electricity consumption^30^. The sociology approach helps to understand the reasons for variations in the consumption behavior of society, with culture, technology, and history^11,47^. The author^48^ discusses social and behavioral aspects of energy use and examines the impact of human behavior on energy consumption. It stresses the importance of incorporating these factors into energy research and policy. This field aims to understand and shape society and individual behaviors for responsible energy use. It underscores the complex connection between society, individual behavior, and energy consumption for a sustainable future^48^. The author^49^ focuses on the environmental impact of human activities in the Anthropocene era. The role of Electric energy in greenhouse gas emissions is highlighted. The organizational Energy Conservation is emphasized to address the ethical and social justice concerns. The energy-saving measures are categorized as "hard" and "soft" interventions.

The study advocates for real-world research to inform future applications and assess the impact of different intervention types and contextual factors on energy efficiency and conservation^49^. The author^50^ has been studying the revelation since 1975, revealing income, age, education, and other factors influencing energy behavior. This supports human Ecosystem theory. The review emphasizes customized energy conservation programs for individuals. It includes motivation, feedback, and indoor air quality considerations. Folk knowledge and adaptation to changing technology are essential. Geopolitical uncertainties must be addressed for sustainable energy practices^50^.

The article^51^ performs a meta-analysis of energy conservation experiments. It shows that these approaches can reduce the use of electricity by 7.4% on average. The study highlights the importance of education, information programs, and technology. Policymakers are encouraged to tackle methodological challenges for better strategies^51^. Technological advancements enable cost reduction and peak demand management through flexible appliances, including electric vehicles. An optimization algorithm allows the consumers to maximize savings and reduce peak-to-average consumption ratios, validated with real-world data from a residential community, achieving a Flattening Index of 0.83. Future enhancements could involve automated optimization using Machine Learning and Internet of Things (IoT) integration^52^. The literature in Table 2 shows the research gap of inadequate understanding of the factors influencing consumer behavior regarding electricity consumption, energy bill evaluation, and optimization, using real data in conventional grid systems. The research question based on the proposed research gap is "How should the conservation of electricity with the reduction of the electricity bill of consumers be achieved?”

This paper addressed the fundamental problem of Maharashtra State Distribution Company Limited. (MSEDCL), utility related to energy bill evaluation in the Indian power sector. Most of the MSEDCL infrastructure for the residential consumer category uses manual meter reading and billing mechanisms. Moreover, the MSEDCL utility also screens and validates bills before bill generation, but only on those bills whose consumption recorded more than four times their average consumption as an abnormal case^5,53^. Due to this high threshold, there are more chances to generate false statements. In this case, consumers can file complaints in a three-tier forum system. It may happen due to large numbers of consumers under one utility and less workforce to handle the issues. Due to the less effective cross-check billing mechanism system, more cases are recorded in the Consumer Grievance Redressal Forum (CGRF). Hence, it reduces consumer satisfaction and trust in utility services^5,53^.

The Maharashtra Electricity Regulatory Commission (MERC) Multi-Year Tariff (MYT) orders and CGRF reports find the gaps in existing billing mechanisms and consumer satisfaction issues^5,53^. Thus, an urgent need is to overcome the billing and metering-related problems and enable the transference in the billing process. On the utility side, if the utility company allows cross-checking recorded units of manual meter reading process with the predicted team at billing software place at the billing department, then the utility can also identify billing errors before generating the electricity bills. Thus, this cross-check system attempts to create transparency in the billing process and build a healthy relationship between consumers and utility companies. The utility can also display the predicted units of the next month before the history unit consumption of twelve months as targeted units on the electricity bill. A utility may also show the unit consumption analysis of the present month analysis through peer comparative analysis. The utility can also resolve cash flow issues through predicted unit prepaid billing on the same meter by giving incentives under the demand response program. It helps to encourage the active participation of consumers and also builds awareness of energy efficiency and energy conservation. MERC also suggested that MSEDCL utility use electricity bills as a tool to create consumer awareness^5,53^. This is the ground-level research work that helps to strengthen the existing electricity infrastructure with the active participation of consumers for the further deployment of innovative grid policies. On the same line, the reviewed literature stated that the problem of false billing, high monthly electricity bills, and faulty meter status of many consumers of MSEDCL utility is in the existing manual meter reading and billing process. The same problem of consumers is addressed through complaints addressed in three-tier consumer forums^5,53^. The significant issues recorded in the three-tier system are consumers getting false, high electricity bills, and installed faulty energy meters. For the same, they are applying the peer comparative electricity unit consumption analysis method. Moreover, this approach helps push individual occupants towards positive action through EE behavioral change^19^. The research gap of no effective cross-check system is to reduce false billing problems in the existing manual meter reading process of MSEDCL utility, from the literature. The research question based on the proposed research gap is, "How do we make a cost-effective cross-check mechanism in the electricity billing process of utility?"

### Problem statement and contributions

This research addresses the challenges in the manual meter reading and billing system of the domestic electricity connections, particularly within MESDCL. The sources of errors in billing systems, both human and machine-related, are identified. The existing energy bill verification system in conventional grid systems is critiqued for its generalized and threshold-based nature, which may lead to inequitable consumer billing^5^. Various nature-inspired algorithms are discussed in^54–58^ for different applications, with less emphasis on household energy conservation. The study proposes a novel methodology for household electricity conservation and energy bill evaluation, introducing a socio-inspired optimization approach, specifically the socio-technical competitive behavior model. This innovative approach incorporates a peer-and self-group competitive behavior paradigm within the socio-inspired optimization framework, paving the way for a knowledge-based decision system to enhance energy conservation and energy bill evaluation. The research thus contributes to bridge the gap in applying socio-inspired optimization approaches for household electricity conservation and energy bill assessments, offering a promising avenue for further exploration in the field^11^. As per literature and knowledge, the proposed SHOEC algorithm and knowledge-based decision-making system for energy bill evaluation and optimization by Socio-inspired optimization methodology is the maiden attempt to use an evolutionary optimization algorithm for household energy conservation^44–46,54–58^.

**Figure 1:**
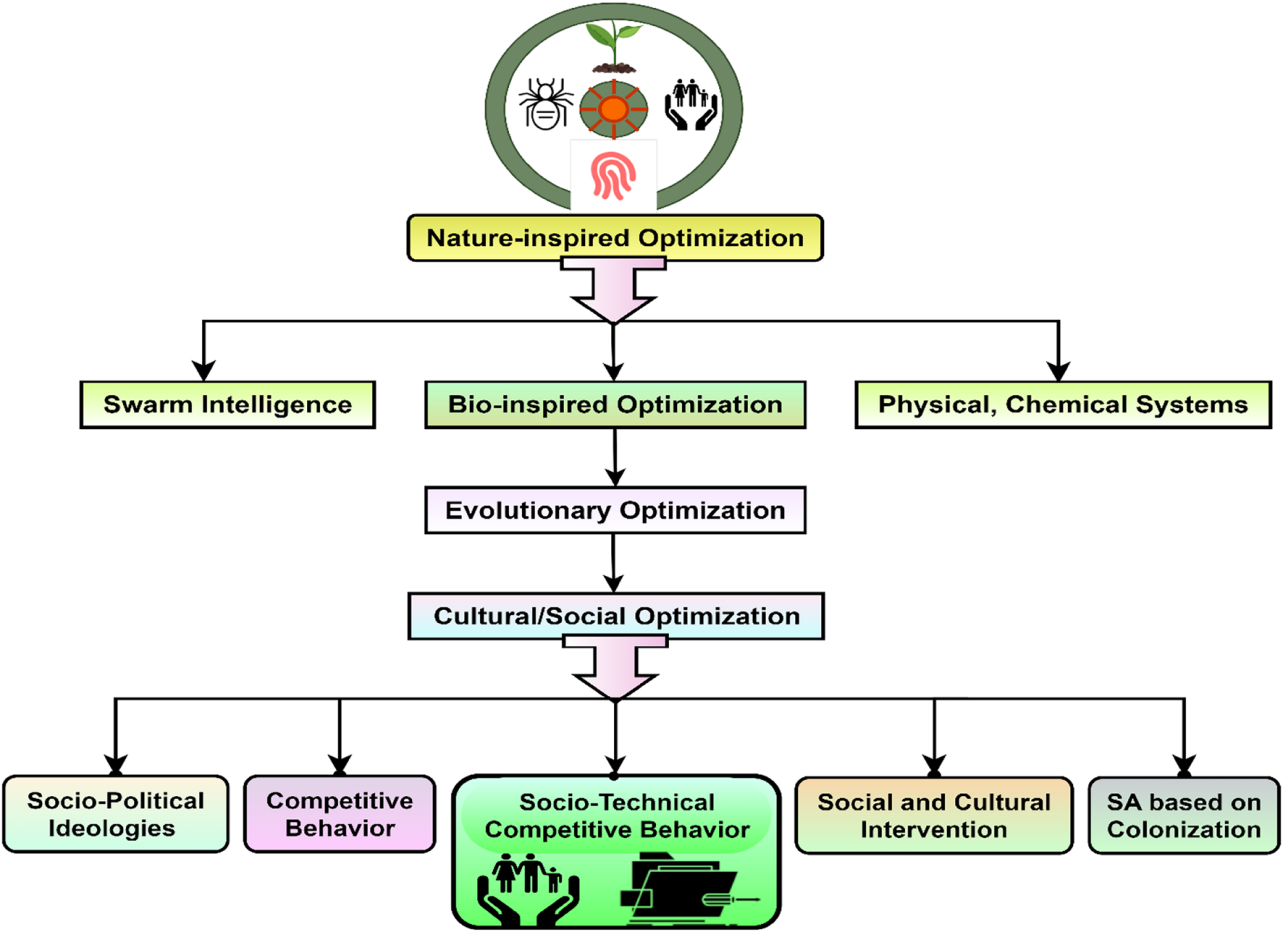
Classification of nature-inspired optimization showing proposed socio-technical competitive behavior approach.

**Figure 2:**
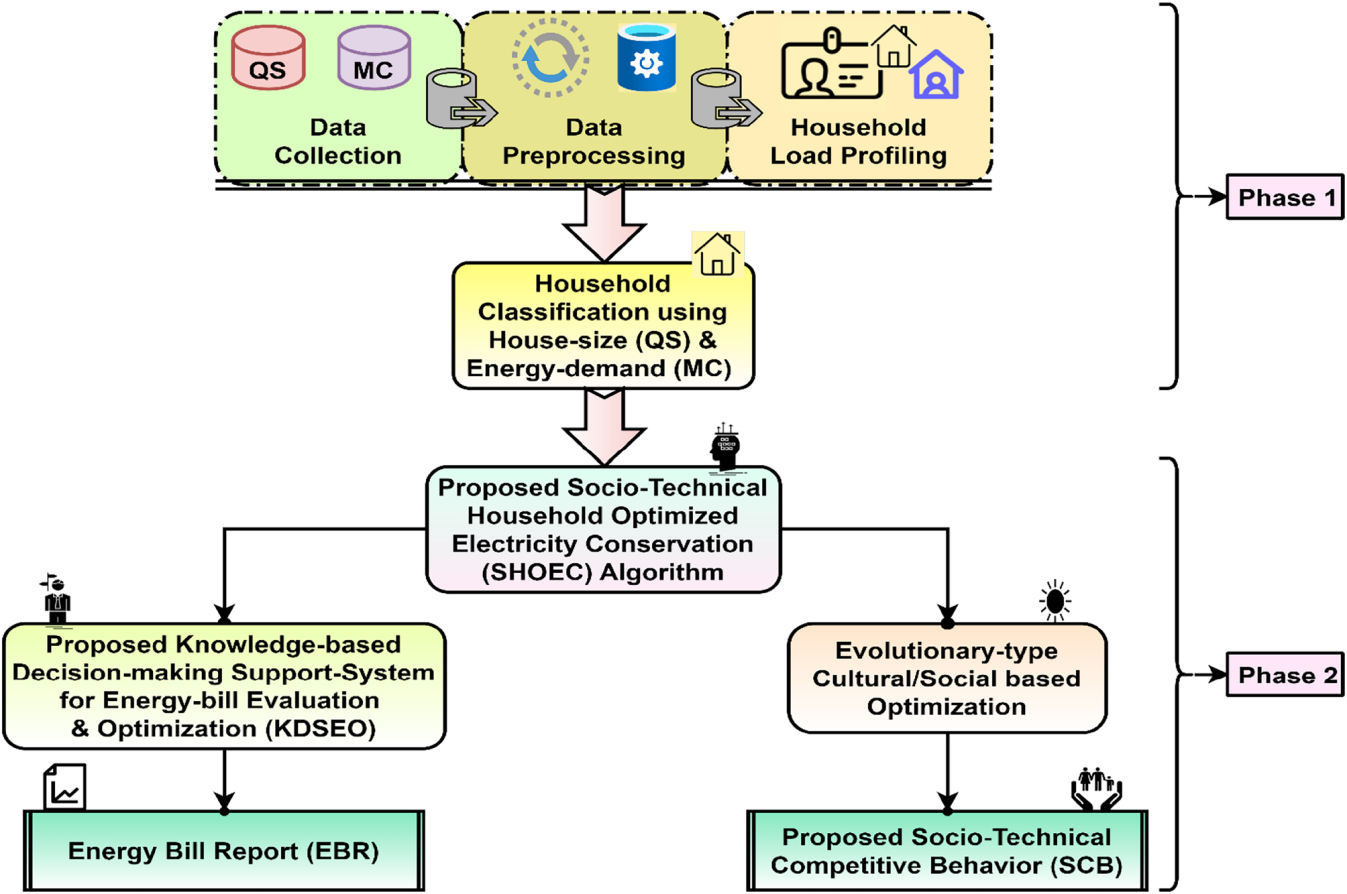
Proposed methodology for household electricity conservation and energy bill optimization. *Note* QS- Questionary survey, MC- Monthly energy consumption.

However, we proposed Socio-Technical Competitive Behavior (SCB) as a new class of Socio-inspired optimization under a Social Algorithm, as shown in Figs. 1, and 2. This problem is defined and addressed by developing a Knowledge-based Decision-making System for Energy bill evaluation and Optimization (KDSEO) using a proposed peer-and self-group learning approach based on the SHOEC algorithm. The proposed KDSEO system aims to generate EBR, providing information to end consumers to evaluate bills and make informed decisions^3^. This approach can help consumers in the following manners:

1. Energy bill assessment (i.e. normal or high)
2. Compare and categorize individual homes based on energy uses: excellent, perfect, and poor in similar home sizes.
3. Identify energy conservation potential in units (kWh) and cost in money using the proposed Socio-technical Household Electricity Conservation algorithm.
4. Develop a knowledge-based decision-making system for EB assessment and optimization.

## Methodology

As per the literature study, the socio-inspired optimization interdisciplinary approach is not sufficiently attempted for household electricity conservation and energy bill evaluation problems in the Indian context on conventional grid systems^1–5,19^.

This is due to its recent evolutionary optimization approach^54–58^. This gap is addressed by proposing a methodology for household electricity conservation and energy bill evaluation. This includes the peer and self-group competitive behavior approach under the SO approach. This approach develops a knowledge-based decision system for energy conservation and energy bill evaluation^4,5^. In addition to this, the proposed work has coined the new category of socio-inspired approach under cultural or social optimization named socio-technical competitive behavior, as shown in Fig. 1.

The proposed work addresses the problem of household electricity manual energy meter reading and billing mechanism of the consumers. Human error and machine error are sources of generating erroneous energy bills. The utility company validates energy bills based on a generalized, threshold-based method that could be more robust. Since household energy consumption is socio-technical, dynamic, and complex, relying on a single fixed rule for validating energy bills is unjustifiable. There is a chance of receiving a high energy bill, possibly due to the usage of the occupants. The knowledge-based system will guide household energy consumers by determining whether the monthly energy bill is normal or high and providing information on possible energy conservation potential in kilowatt-hours (kWh) and cost in rupees. Figure 2, shows the proposed methodology for a knowledge-based decision-making system for energy bill evaluation and optimization for the domestic consumer.

The proposed method is divided into two subsections in Phases:

### Phase 1: Data collection, preprocessing, load profiling, and household classification

In this phase, four components are included: Data collection, preprocessing, household classification, and household load profiling. These components are discussed in detail in a previously published paper^1–3^. A brief overview of the process is discussed in this section.

This study used different datasets, namely QS, MC, and tariff order, which contain data from 225 consumers from Pune, Nashik, and Ahmednagar districts of Maharashtra, India. The MC dataset includes 40 months of energy consumption from January 2019 to April 2022. The study used a structured Questionnaire-based sample survey data collection with a Random Sampling technique. The proposed research with a questionnaire survey was presented to the Independent Ethics Committee (IEC), Symbiosis International (Deemed University), Pune-412115, India. IEC has approved the study and has stated that no direct human participation or ethical issues were found. The informed consent was obtained from all the study participants. This study methodology and objectives are also approved by the Technical Institutional Research Advisory Committee of Symbiosis International (Deemed University). So, all methods were carried out according to relevant guidelines and regulations. The complete questionnaire is available in the Appendix section.

**Figure 3:**
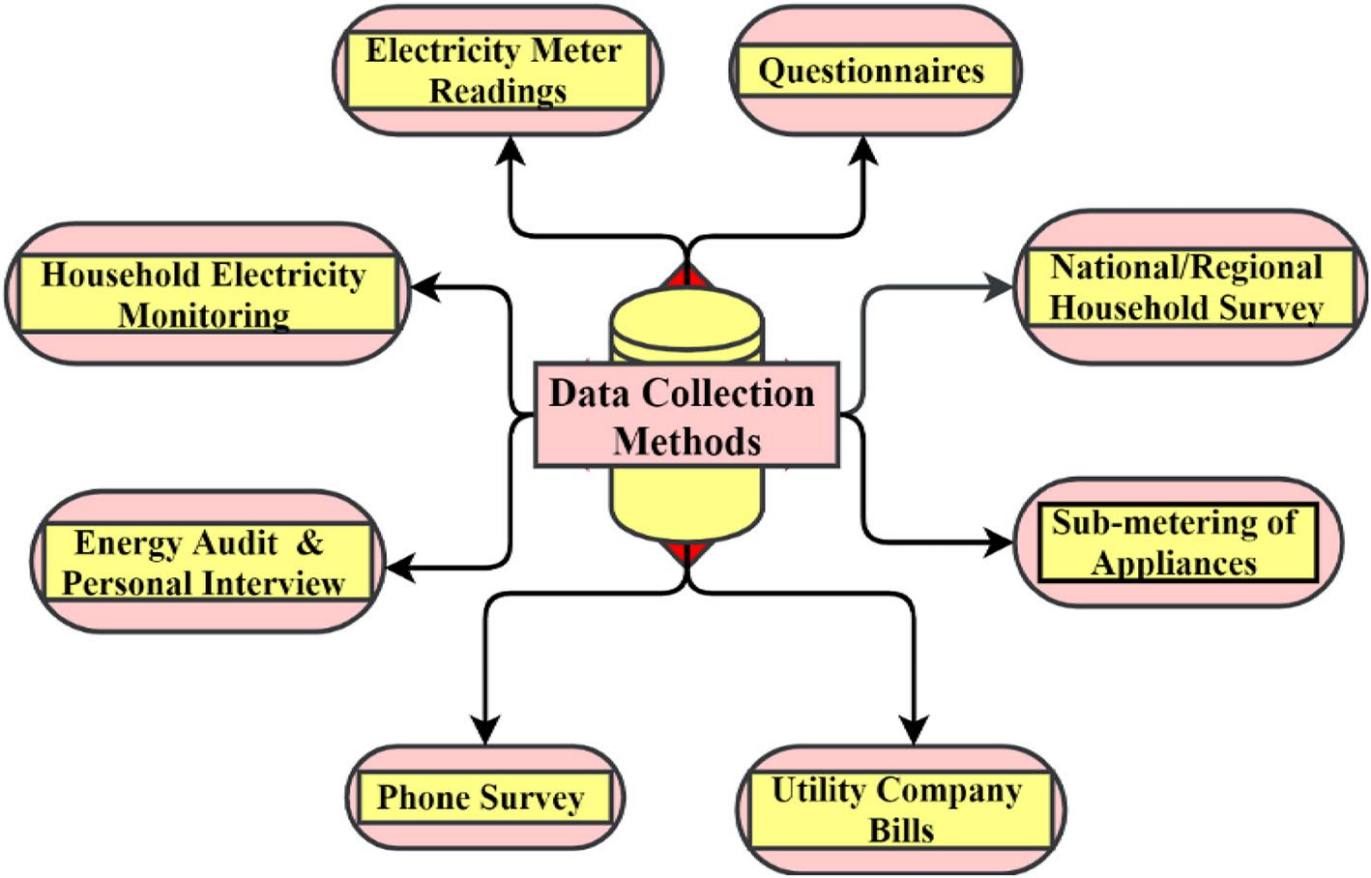
Methods of data collection.

According to the literature, various data collection ways are available for household electricity consumption studies, namely personal interviews, telephone surveys, energy meter readings, HEC pattern monitoring, individual appliance consumption using sub-meter, Questionnaires-based surveys, energy audits, National or Regional level household surveys, and monthly bills of the ESP company as shown in Fig. 3^1,2,21^. The structured questionnaires-based survey method is used to collect household electricity consumption-related data. Generally, the prerequisite step is the first to define the problem statement and design the questionnaire survey discussed in Section “Problem statement and contributions”^1,3^.

The proposed study in the Indian context has not been attempted earlier. Hence, the authors prepared the questionnaire as per the problem statement. Due to multiple factors (direct and indirect) affecting the dynamic nature of residential energy consumption, we could not directly include existing questions from the literature. However, they were referred while preparing the questionnaire. It also involved various energy utility companies, Engineers, academicians, and researchers from the same domain. Moreover, inputs from the Independent Ethics Committee and Research Advisor Committee were also considered.

Moreover, this questionnaire survey form is communicated to the participants on the online platform via Google Form through email, social media, and offline method through provision of a hard copy of the questionnaire. It is observed that the response rate for online communication questionnaire survey forms through email or social media was honored to be lower^1^.

The main reason is that the detailed study includes 34 quantitative and multiple-choice questions^1,3^. To increase the rate of the response the on-site field visit, and offline hardcopy-based data collection approach are used. The specialties of the designed Online Questionnaire survey Google Form include: added conditional type or dependent type of question (skipped the six questions if uploaded the image of electricity bill), added photos (type of appliances, tree shade, water bodies, Earth Leakage Circuit Barker, switching off primary devices, a symbol of Star label etc.), provided questions in two languages (English and regional Marathi), applied input Validation method through various formulas and threshold or range values based on pilot survey responses for avoiding garbage responses^1–3^.

**Figure 4:**
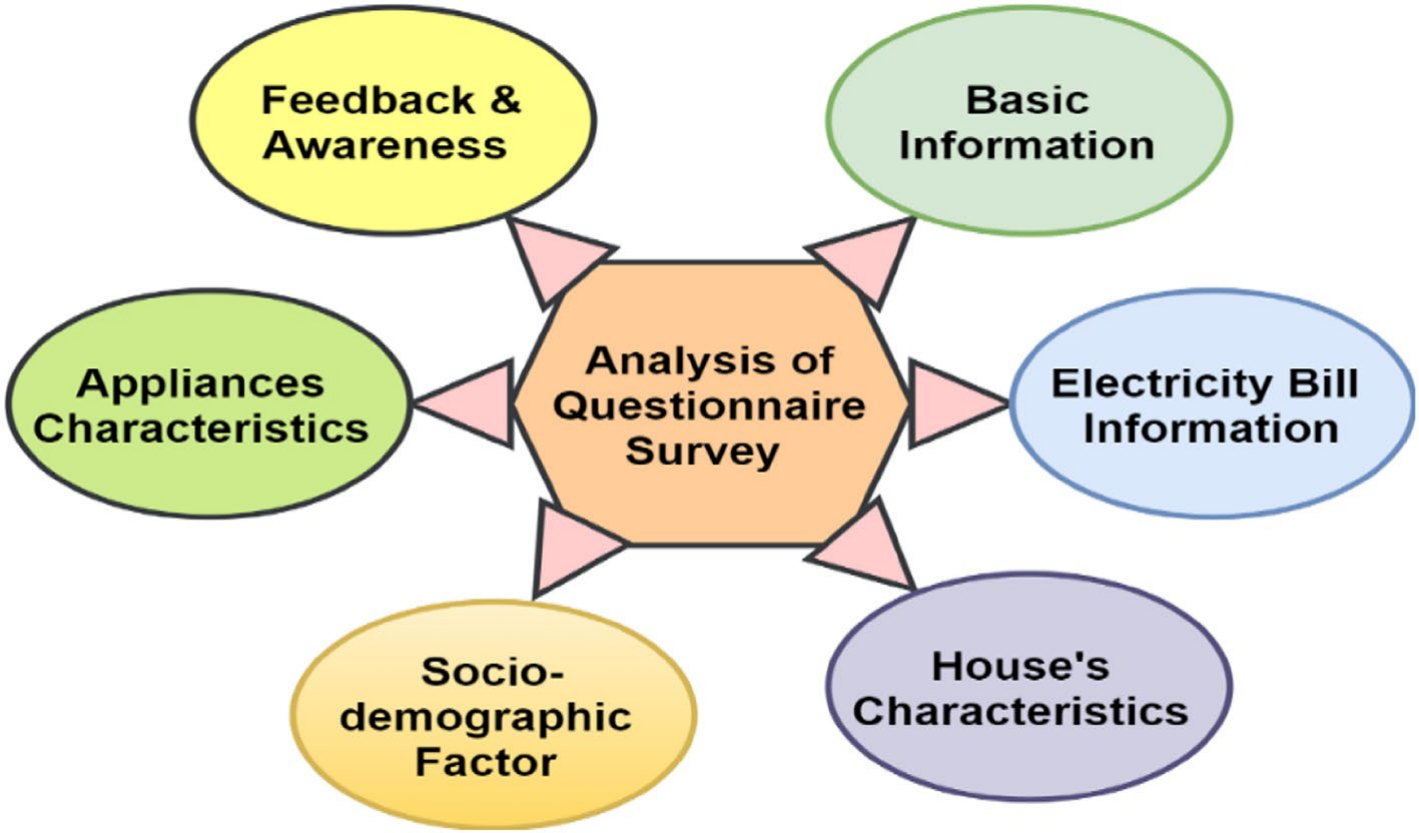
Elements of a questionnaire survey.

Furthermore, the survey form is broadly divided into six elements: basic information, electricity bill information, house characteristics, socio-demographic factors, appliance characteristics, feedback, and awareness, as shown in Fig. 4. Table 3, shows the five categories of QS with different variables on which questions are drafted^1–4^. Feature Engineering (FE) data preprocessing techniques add new essential features based on the QS and MC datasets. Based on the QS dataset, six important parts are generated: the sum of socio-demographic parameters, total regular equipment, total lifestyle equipment, number of five-year-old equipment, the sum of QS (QsEqu), and the sum of essential factors (QsSum).

**Table 3:** List of categories in QS with variable.

Sr. no	Categories	Variables
1	Socio-demographic	Monthly income, number of people living in the house, education level and geographical location
2	House characteristics	Type of house, tenure type, home location in the building, housing carpet area, number of rooms, number of windows, number of balconies, facing of home door direction, living years in home
3	Appliance characteristics	Total number of appliances, set temperature for the air conditioners, Earth Leakage Circuit Breaker (ELCB), more than five years old, star-labeled appliances
4	Electricity consumption	History unit consumption, meter status, source of power supply to the home, type of power outage, voltage fluctuation
5	Feedback and awareness	Ventilation and sun-lighting: turn off your major household appliances from switchboards (stand-by consumption), tree shade, and water bodies

Similarly, the MC dataset has added four critical features: peak consumption, standard deviation, monthly sum, and average monthly energy consumption (Avg_kWh). Household classification is essential to understand the similarity among the houses. The household classification, with its prediction study, contributes to energy conservation. The QsSum and Avg_kWh attributes are used separately for optimal home classification. QsSum classifies the houses into four groups: Small Houses (SH), Medium Houses (MH), Large Houses (LH), and Very Large Houses (VLH). Similarly, the Avg_kWh attribute classifies households into four groups, namely Household 1 (small house with low energy demand), Household 2 (Medium house with medium energy demand), Household 3 (Large house with peak energy demand), and Household 4 (Very large house with peak energy demand)^7^. This classification is based on an expert-based classification method and uses K-Means, Hierarchical, and Self Organizing Map (SOM) clustering with direct, indirect, and multistage techniques. The 2nd stage, K-Means with SOM indirect clustering, has provided better clusters to classify the households^1–4^. Further, the classification of both the families is compared to find the hidden consumption patterns of the homes. Thus, optimal house classification is necessary for monthly energy consumption prediction and EB optimization.

### Phase 2: household energy consumption prediction and KDSEO for energy bill

This subsection includes the majority of the proposed work, namely monthly prediction, Socio-Technical Household Optimized Electricity Conservation (SHOEC) algorithm, to develop a Knowledge-based Decision-making Support system for EB Evaluation and Optimization (KDSEO) and to generate Energy Bill Report (EBR) for energy bill evaluation as shown in Fig. 2. The output of household classification based on similar house size (QS) and energy consumption (MC) is used as input to the household monthly energy consumption prediction model, for this proposed hybrid prediction model is called SMSDAR^3,4^. The SMSDAR prediction model contains a Support Vector Machine (S), Multi-layer perceptron (M), Stochastic Gradient Descent (S), Decision tree (D), Adaptive boosting (A), and Random Forest (R). This model has improved the prediction accuracy up to 92% from the highest accuracy of 76% of the individual from individual algorithms. The monthly prediction is one of the inputs required for self-group consumption analysis for the proposed Socio-Technical Household Optimized Electricity Conservation (SHOEC) algorithm, as shown in Fig. 2. The human behavior tendency of comparison between peer and self-groups based on the socio-technical competitive behavior approach is used for the SHOEC. Furthermore, a knowledge-based decision-making system for energy bill evaluation and optimization system is developed, using competitive energy consumption analysis for energy savings. The motivation is to use a nature-inspired algorithm, that is cultural or social optimization. The human behavior tendency of comparison between peer and self-groups based on the socio-technical competitive behavior approach is utilized to design a knowledge-based decision-making system, as shown in Figs. 1 and 2.

- Methodology testing approach with mathematical modeling of the KDSEO system

**Figure 5:**
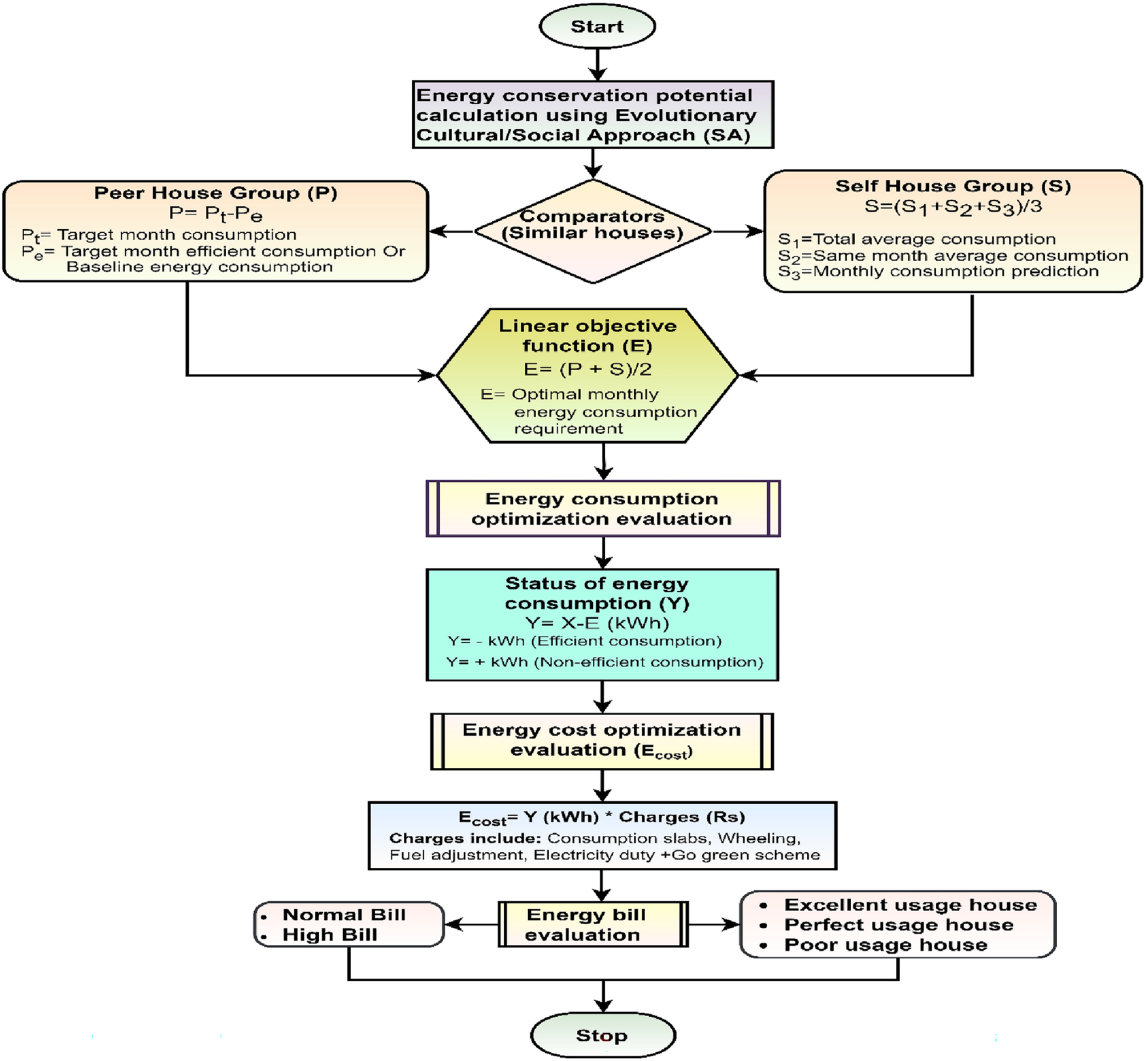
Testing flowchart for proposed methodology with KDSEO system.

The Socio-Technical Household Optimized Electricity Conservation (SHOEC) algorithm aims to find the optimal energy conservation potential of each household in kWh and cost in Rupees as shown in Figs. 2 and 5. SHOEC algorithm used two key parameters: Peer (P) and Self (S) comparators. Due to this parameter, a SHOEC belongs to the Cultural or Social Algorithm. The proposed algorithm is part of SO due to the consideration of the critical parameters such as Peer (P) and Self (S) comparators. Furthermore, various features are supposed to develop a knowledge-based decision-making system for energy bill evaluation and optimization under P and S comparators as shown in Figs. 2 and 5. P comparator needs similar house-size homes to compare monthly energy consumption. The minimum EC potential of each house has been calculated based on a comparative approach. To achieve this purpose, low energy consumption in similar house sizes is considered baseline consumption or efficient house. The difference between baseline consumption and similar houses will give the peer-based minimum EC potential. Equation (1) shows the mathematical representation of the P comparator in kWh.

1 $$P_{i} = {\text{P}}_{{{\text{ti}}}} - P_{ei}$$

where *Pi* is the EC potential of the ith house, Pti is the energy consumption of the target month of the ith house, and ${P}_{ei}$ is the energy consumption of an efficient house of similar size. Similarly, the S comparator finds minimum energy conservation potential of each household. The S is calculated based on three parameters, namely the average energy consumption of each similar house class (S1), an average of the last years of same-month energy consumption (S2), and the monthly energy consumption prediction of each house (S3). First, S1, S2, and S3 are used to find the separate EC potential by taking the difference with target month energy consumption termed S_1i_, S_2i_, and S_3i_.

2 $$S_{1i} = S_{1ti} - S_{1b}$$

where ${S}_{1i}$ is the EC potential of the ith house of S1, *S*1*ti* is the ith house's target monthly energy consumption of S1, and ${S}_{1b}$ is the baseline energy consumption of each house class as the average of a whole class of S_1_.

3 $$S_{2i} = S_{2ti} - S_{2si}$$

Where ${S}_{2i}$ is the EC potential of the ith house of S_2._, ${S}_{2ti}$ is the ith house target monthly energy consumption of S_2_, and ${S}_{2si}$ is the same target month average energy consumption of each house of whole class of S_2._

4 $$S_{3i} = S_{3ti} - S_{3pi}$$

where ${S}_{3i}$ is the EC potential of the ith house of S_3._, ${S}_{3ti}$ is the ith house target monthly energy consumption of S_3,_ and ${S}_{3pi}$ is the monthly energy consumption prediction of each house class as the average of whole class of S_3_. Finally, the minimum EC potential (S) is calculated by taking an average of each EC potential of ${S}_{1i}$, ${S}_{2i}$, and ${S}_{3i}$ from Eqs. (2)–(4), as given in Eq. (5)

5 $$S_{i} = \frac{{S_{1i} + S_{2i} + S_{3i} }}{3}$$

The EC of household consumers depends on multiple dynamic and complex factors. Thus, to find the optimal monthly EC potential of an individual house, the average of ${P}_{i}$ and ${S}_{i}$ values from Eqs. (1) and (5) are considered.

6 $$E_{i} = \frac{{P_{i} + s_{i} }}{2}$$

where ${E}_{i}$ Indicates the linear program objective function for optimizing the i^th^ house EC potential, the ${E}_{i}$ value is used further to find the expected monthly energy consumption (${Y}_{i}$) as shown in Eq. (7).

7 $$Y_{i} { } = X_{i} { } - E_{i}$$

where ${X}_{i}$ is the actual monthly energy consumption from EB, the value of E and Y is critical to motivating households for EC and Optimization. These attributes are further used to find the EC regarding the rupees (Rs) cost. Additionally, how efficiently has each household used the MC. The MC has been calculated by considering consumptions like actual MC and Y separately, as shown in Eqs. (8) and (9).

8 $$A_{i } \% = \left( {\frac{{E_{i} }}{{X_{i} }}} \right) *100$$

where ${A}_{i}$ is the EE of *i*th house respective to actual consumption ${X}_{i}$ This equation provides information on the % extra used energy during the month. Similarly, ${B}_{i}$ is the EE of *i*th house respective to expected monthly energy consumption (${Y}_{i}$) in percentage as shown in Eq. (9).

9 $$B_{i} \% { } = \left( {\frac{{E_{i} }}{{Y_{i} }}} \right) *100$$

The optimal EE is calculated by taking an average of Eqs. (8) and (9) termed as ${Z}_{i}$ in percentage in Eq. (10):

10 $${ }Z_{i} \% = \left( {\frac{{A_{i} + B_{i} }}{2}} \right)*100$$

where ${Z}_{i}$ is the monthly optimal EE obtained for the *i*th house in percentage. The sample results of Eqs. (6)–(10) are mentioned in Table 6.

- Linear programming and Genetic algorithm approach for energy conservation analysis

**Figure 6:**
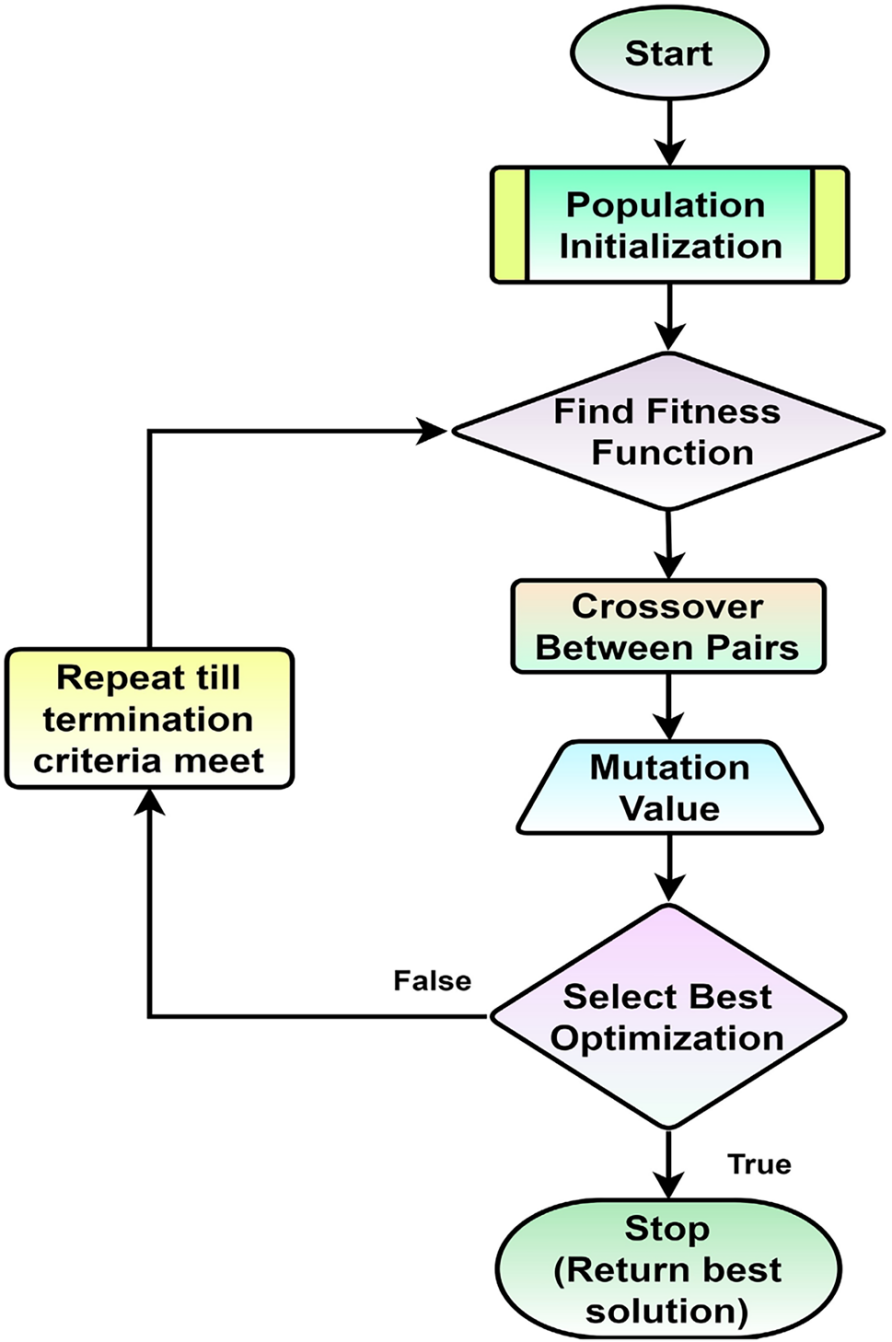
GA approach for household energy conservation.

Linear programming and GA optimize energy consumption for household categories based on socio-demographic constraints. This will help to analyze self and peer-group consumption behavior based on socio-demographic constraints. This will also help the consumers with electricity bill evaluation range (i.e., normal, high, or very high) and find the energy conservation potential (kWh) and cost savings to solve complex real-world electricity conservation problems. Figure 6, shows the flowchart for GA approach testing with Linear Programming. The first step is to create the population and then fit the chromosomes in the selection process. The crossover between pairs is used as one of the parameters. This helps to maintain that the probability lies between 0 and 1; based on the trial method, the best results are achieved at 0. Similarly, based on the trial method, population size and termination criteria are structured^12,13^. This ensures that the Genetic Algorithm focuses on the best candidate solutions. In the crossover process, the two parent chromosomes combine to form a new daughter chromosome, which inherits specific characteristics from each of its parents.

This process helps to create new candidate solutions that differ from the original population. The process continues until the best solutions are attained or the number of iterations is completed^54,55^. Here, different house category-based objective function is designed.

Other populations of room size, number of appliances, and members are used to find the fitness of the objective function. The best solution-based population is selected, and the worst are discarded, as shown in Tables 9 and 10. The initial population is 100, and the fitness value based on the two worst solutions is discarded^12,13^. The rest of the population is paired in a group of two for crossover, and then the mutation is performed to flip some random value for a better population. After this step, two random solution populations are again inserted, and the process is continuous until the conversion criteria are met or several iterations are completed. At this point, the best-fit point provides the optimal energy consumer^55,56^.

Basic outlines involved in Genetic Algorithm are presented below with the example^12,13^:

1. Define the problem and the objective function: Here, house category-wise objective functions are defined, and the constrained Genetic Algorithm problem is formulated, so the constraints are also mentioned in Tables 9 and 10.
2. Generate an initial population of candidate solutions: Each solution is represented by a chromosome, which encodes the variables in the equation. So, a total of 100 populations are created based on the random values of the parameters such as the Number of Rooms (R), Appliances (A), and family Members (M) in the house.
3. Evaluate the fitness of each individual in the population by calculating the value of the objective function: Here, fitness values are calculated for 100 populations, the worst two solutions are discarded, and the rest are paired for crossover, as shown in Eqs. (11–13)^59,60^.
  11 $$Fitness = Maximum\;objective\;function\;value - individual\;member\;function\;value$$
  12 $$Probability\;addition = \frac{Cumulative\;sum\;of\;fitness}{{sum\;of\;fitness}}$$
  13 $$Chromosome\;Selection = sum\left[ {(Prob. addition*ones\left( {1, Pop. size} \right) < ones\left( {pop. Size,1} \right)*rand\left( {1, Pop.Size} \right)} \right] + 1$$
4. Create new individuals by applying crossover and mutation operators to the individuals in the mating pool: Based on the crossover and mutation probability, which is considered 0.5 (randomly selected between 0 and 1) to generate the best chromosomes/generations provided by Eqs. (14) and (15)^59,60^.
  14 $${\text{Pairs}}\;{\text{to}}\;{\text{cross }} = {\text{Rand }}\left( {{\text{Pair}}\;{\text{No}}.,1} \right) < {\text{Crossover}}\;{\text{Prob}}.{ }$$
  15 $${\text{Rand }}\left( {{\text{Pop}}\;{\text{Size}}} \right) < {\text{Probability }}\left( {{\text{mutation}}} \right)$$
5. Evaluate the fitness of the new individuals.
6. Repeat steps 3–5 until a satisfactory solution is found or a maximum number of iterations is reached, as shown in Fig. 6.

## Results

A household electricity consumers' manual energy meter reading and billing mechanism problem is addressed in this proposed work. This problem is identified and deployed by using the SHOEC algorithm. A knowledge-based decision-making support system is proposed to consumers for their household energy bill evaluation and conservation. The input required for this decision-making system is a questionnaire survey and monthly household consumers' monthly energy consumption data of consumer. Equations (1)–(10) shows the mathematical aspect of the SHOEC algorithm. Around 225 households' electricity real datasets are used, namely a Questionary survey, monthly energy consumption tariff order from utility companies, and a Knowledge-based decision-making system that provides the energy bill report as the output of this system. The information provides the status of monthly energy bills belonging to the efficient households, normal consumption or high consumption, or error bill categories based on self and peer comparative nature-inspired optimization approach. The consumers cannot simply compare their energy bills with neighbors, and now allow them to make informed decisions based on the provided report. The methodology Section discusses all proposed methodologies, from input data collection to the outcome.

### Data description

**Figure 7:**
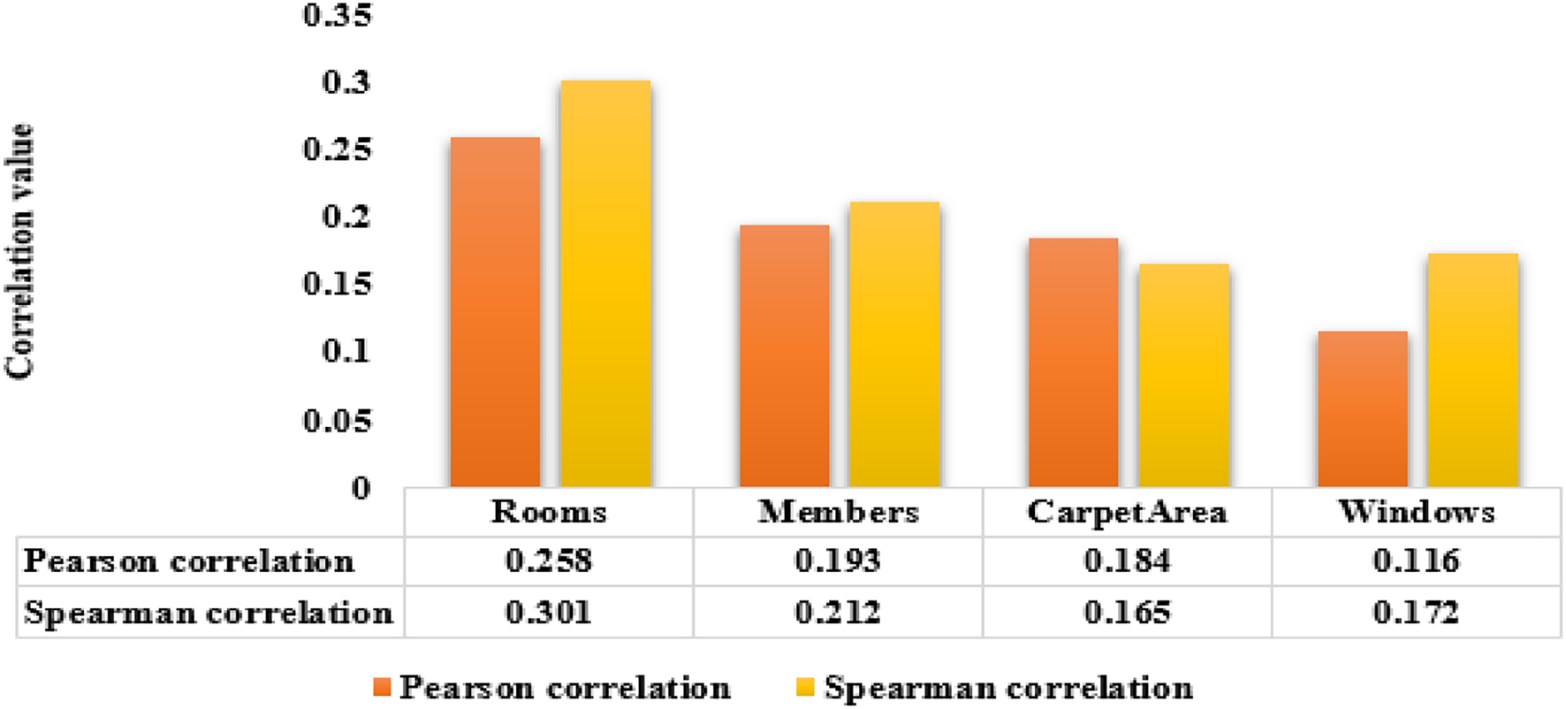
Correlation analysis of selected household characteristics with the energy consumption.

The dataset of 225 households is collected from three districts of Maharashtra, India. Three datasets, namely QS, MEC, and tariff orders, are collected. The QS is primary data, and MEC and tariff orders are secondary data, but they cannot be used directly due to various data quality issues and understanding. Different data preprocessing techniques are used to improve the data quality, like identification and imputation for missing values and outliers, data integration, converting categorical variables, handling varying and irrelevant data, text cleaning, feature engineering, data validation, and data quality assessment^1–4^. This method, including Feature engineering, is discussed in the methodology Section. The existing significant features of dataset are identified using Spearman (S) and Pearson (P) correlation methods respective to average monthly energy consumption in kWh. Figure 7 shows the S and P correlation-based significant features: the number of rooms, family members, carpet area, and windows. The carpet area is the actual living or working space of the house measured square feet. Identification of significant factors in energy consumption is essential for further study of household consumer classification and prediction model analysis.

### Knowledge-based decision-making system analysis

After data collection, methods and techniques like data preprocessing, feature engineering, clustering, classification, and prediction modeling were implemented in previous work^1–4^. The next step is to develop a Knowledge-based Decision-making System for energy bill Evaluation and Optimization (KDSEO) of competitive energy consumption analysis as energy savings. The aim is to use a nature-inspired algorithm, that is cultural or social optimization. The human behavior tendency of comparison between peer and self-groups based on the Socio-technical Competitive Behavior (SCB) approach is utilized to design a knowledge-based decision-making system. The socio-technical comparative behavioral approach is used to develop the Socio-Technical Household Optimized Electricity Conservation (SHOEC) algorithm. Equations (1)–(10) shows the mathematical modeling of the proposed SHOEC algorithm. However, Fig. 5 shows the testing flowchart for the proposed methodology with the KDSEO system, including the SHOEC algorithm. Table 4, shows the features used in the SHOEC algorithm and the attributes name of the energy bill report.

**Table 4:** Model notation of energy bill report.

Sr. no.	Parameters	Description	Assigned code
1	HID	Household Identification	F1
2	QS_Equ_Sum	Sum of significant factors of QS	F2
3	EM_Status	Energy meter status	F3
	3.1 NMR	Normal meter reading	F3
	3.2 AMR	Average meter reading	F3
4	GoG_Status	Go Green Scheme Status	F4
	4.1 NOP	Non-opted GoG	F4
	4.2 OP	Opted GoG	F4
5	Total_Avg_kWh	Average energy consumption	F5
6	LYsSM	Average consumption of same months	F6
7	MPD	Monthly energy consumption prediction	F7
8	Xi	Target month energy consumption of ith house	F8
9	Yi	Expected MC of target month of ith house	F9
10	Ei	Minimum EC potential of ith house	F10
11	Profit_Rs	Minimum cost saving potential in Rupees	F11
12	Zi %	EE of ith house in percentage	F12
13	EB_Status	Energy bill type for the month	F13
	13.1 NB	Normal bill	F14
	13.2 HB	High bill	F14
14	House_Usage_Category	Household classification based on usage	F14
	14.1 EUH	Excellent usage house	F14
	14.2 PeUH	Perfect usage house	F14
	14.3 PoUH	Poor usage house	F14
15	Action_Group	Experimental and control groups	F15
	15.1 CG	Control group	F15
	15.2 EG	Experimental group	F15

The inputs for the proposed Knowledge-based decision-making system are based on the output of the prediction model and by considering self and peer parameter comparison. The critical parameters for the optimization model are self and peer-comparative analysis. The input of the Knowledge-based decision-making system includes household electricity consumer classifications and monthly energy prediction as one of the parameters, and its assigned code is F6, as shown in Table 4. F1–F15, except for F6, are the parameters used to develop the proposed Socio-technical Household-Optimized Electricity Conservation (SHOEC) algorithm. The critical parameters for SHOEC are self-based and peer-based competitive behavior parameters. Table 5, shows fifteen major parameters with the assigned codes from F1 to F15 to develop a knowledge-based decision-making system for energy bill evaluation and conservation. The model notation of the sample electricity bill report is the outcome of the decision-making system with the parameters used, as shown in Table 5^55–61^.

**Table 5:** Sample electricity bill report (EBR) as the final output for end consumers.

House_Type	F1	F2	F3	F4	F5	F6	F7	F8	F9	F10	F11	F12	F13	F14	F15
Small House	129	35	NMR	NOP	23	32	22	18	25	− 7	− 34	− 69	NB	EUH	CG
	117	44	NMR	OP	33	36	64	69	42	27	51	120	HB	PoUH	EG
	181	44	AMR	NOP	38	37	48	37	40	− 3	− 7	− 20	NB	EUH	CG
	49	45	NMR	NOP	32	39	44	49	45	4	8	51	NB	PoUH	EG
	132	45	NMR	NOP	29	28	52	62	45	17	33	126	HB	PoUH	EG
Medium House	187	52	NMR	NOP	83	68	106	117	87	30	30	243	HB	PoUH	EG
	149	66	NMR	NOP	67	50	46	80	75	5	7	36	NB	PeUH	CG
	64	63	NMR	OP	46	36	61	58	58	0	0	− 1	NB	PeUH	CG
	147	60	NMR	NOP	61	68	84	9	37	− 28	− 194	− 142	NB	EUH	CG
Large House	155	80	NMR	NOP	145	144	204	240	95	145	106	1366	HB	PoUH	EG
	119	85	AMR	NOP	211	178	221	203	82	121	104	1089	HB	PoUH	EG
	111	71	NMR	OP	151	185	142	141	150	− 9	− 7	− 78	NB	EUH	CG
	25	74	NMR	NOP	136	189	200	97	140	− 43	− 38	− 818	NB	EUH	CG
Very Large House	108	113	NMR	NOP	279	188	396	405	357	48	13	378	HB	PoUH	EG
	208	110	NMR	NOP	287	330	285	276	297	− 21	− 8	− 144	NB	EUH	CG

The house type categories are formed based on similar household size and energy consumption patterns. The household categories are small house, medium house, large house, and very large house categories. The similarity-based energy bill information is essential for consumers to make an informed decision on their energy bill evaluation, overcoming unthinkingly comparing the energy bill with a Neighboring household. The significant attributes used for self and peer comparative analysis under socio-technical household optimized electricity conservation algorithm are listed as namely, Household Identification (HID) is a unique number assigned to each house, Summation of significant attributes of QS (QsSum), Average energy consumption (Avg_kWh), Average of same month energy consumption of last year (LYsSM), Monthly energy consumption prediction (MPD), April month actual energy consumption (April_kWh), Expected April month energy consumption (Exp_April_kWh), Minimum Energy Conservation Potential (Min_EC_Pot_kWh), Minimum extra energy usage (Extra_Usage_%), Minimum cost saving potential in rupees (Min_Save_Pot_Rs), April month energy bill type (EB_Status_April), April month energy meter status (EM_Status_April), Go Green Scheme Status (GoG_Status), house categorization based on energy consumption in kWh (House_Usage_Class), Group the houses as experimental and control group for further actions (Action_Group).

Further, the minimum energy conservation potential Ei parameter (F10) is used by the tariff order to determine the minimum cost saving potential in rupees Profit_Rs parameter (F11) by considering the slab rate and Go Green Scheme Status GoG_Status parameter (F4) (a rebate of Rs.10/- for online EB copy) as shown in Table 4. Based on the Expected MC of the target month of ith house parameter Yi (F9), the Minimum EC potential of ith house parameter Ei (F10) and energy efficiency of ith house in percentage Zi % parameter (F12) energy bill is evaluated as a normal, high, or defective bill under the EB_Status parameter (F13). Thus, energy bill information is helpful to household consumers in making informed decisions and optimizing EB. Consumers can compare and analyze their monthly EB using fifteen parameters, as shown in Table 5.

Table 6, provides the brief information to evaluate energy bills with limited parameters. This shows the sample results for Eqs. (6)–(10), which give the status of the energy conservation potential of individual households among similar households. The negative sign shows better energy consumption. The highest negative consumption in kWh can be the most efficient house among them. Besides, the positive signed consumption shows the possible energy conservation potential. Further, the values of the Minimum EC potential of the ith house parameter (F10), the expected monthly energy consumption of target month of ith house parameter (F9) and energy efficiency of ith house in percentage (F12) are taken into consideration to classify the home based on usages House_Usage_Category parameter (F14), like Poor Usage House (PoUH), Perfect Usage House (PeUH), and Excellent Usage House (EUH). Based on the Household classification based on the usage parameter House_Usage_Category (F14), the houses are further classified into experimental and control groups under the Action_Group parameter (F15). The action group is additionally required to do continuous monitor consumption and help reduce the energy bill as a future scope of this work. The control group, consisting of PeUH, EUH, and reaming, will be considered the experiment group. There is the scope for improvement in energy efficiency and conservation in the experimental group.

**Table 6:** Sample results for Eqs. (6)–(10) measured in kWh.

F1 (i)	F2	F6	F9 ($Y_{i }$)	F8 ($X_{i }$)	F10 ($E_{i} \%$)	($A_{i }$)	($B_{i} \% )$	F12 ($Z_{i} { }\%$)	Usage_Range%
75	45	27	33	24	− 9	− 38	− 27	− 32	− 38 to − 27
48	43	32	26	18	− 8	− 44	− 31	− 38	− 44 to − 31
13	43	8	21	24	3	13	14	13	13 to 14
123	39	36	54	69	15	22	28	25	22 to 28

Furthermore, the experimental results provide that the information of total 225 houses was considered, and the final dataset included data from 139 homes to develop a decision-making system for energy bills. Figure 5, shows the test flow of the proposed Methodology, Eqs. (1)–(10). The sample results mentioned in Tables 5 and 6 are interlinked and clarify the step-wise process and obtained results. The thresholds were fixed to have variations in the actual and expected consumption based on house sizes, such as small houses, medium houses, large houses, and very large houses. The findings of this study indicate that approximately 31% of bills are categorized as high bills, while 69% are considered normal bills. This shows that the manual meter reading and billing mechanism in conventional grid systems faces the challenge of addressing high bills and exploring opportunities for energy conservation. Sometimes, the high bill may have been generated because of high consumption or technical or human error, etc. The energy bill report of the decision-making system guides consumers to make an informed decision based on the provided fifteen parameters (F1–F15), as shown in Table 5.

### Thumb rule-based energy bill evaluation

Sometimes, due to technical terms in the energy bill report, all consumers need help understanding the energy bill. At the same time, the self-explainability of the provided result can improve the understanding of the energy bill report. The decision-making system may become complex due to the consideration of multiple factors.

This problem is addressed by proposing a thumb rule with less significant Factors which everyone can understand and relate to consumption. They are affecting monthly energy consumption. The thumb rule or generalized rules can also simplify the energy bill evaluation. This pattern of energy consumption range in association with the significant factors is observed from the SHOEC dataset and it also helped to interpret the energy bill report. Tables 7 and 8 help to understand the important factors with the quantity to know the tentative energy consumption range and house type. Thus, the proposed SHOEC algorithm-based energy bill report and thumb rule-based model explainability can provide the knowledge for decision-making for such an uncertain problem.

**Table 7:** Proposed system explainability using thumb rules for Type A house category.

TH	CA (Sq.Ft.)	R	TRA	TLA	TA	5Y + A	M	QS_Equ	Avg_kWh	HT
In_0	In_1	In_2	In_3	In_4	In_5	In_6	In_7	Out_1	Out_2	Out_3
9	100–600	0- 4	0–10	0–4	0–14	0–2	0–3	0–43	0–20	SH
26	701–850	4–5	11–17	5–8	15–25	3–4	3–4	44–60	21–50	MH
21	851–1200	6–7	18–21	9–13	26–34	5–6	4–5	61–75	51–100	LH
2	1200 +	7 +	21 +	13 +	34 +	6 +	5 +	75–111	101–279	VLH

*TH* total houses, *CA* carpet area, *R* rooms, *TRA* total regular appliances, *TLA* total lifestyle appliances, *TA* total appliances, *5Y* + *A* five-year-old appliances, *M* members, *QS_Equ* questionary survey equation, *Avg_kWh* average consumption, *HT* house type, *SD* standard deviation, *SM* small house, *MH* medium house, *LH* large house, *VLH* very large house.

**Table 8:** Proposed system explainability using thumb rules for Type B house category.

TH	CA (Sq.Ft.)	R	TRA	TLA	TA	5Y + A	M	QS_Equ	Avg_kWh	HT
In_0	In_1	In_2	In_3	In_4	In_5	In_6	In_7	Out_1	Out_2	Out_3
20	100–700	0–4	0–12	0–5	0–18	0–2	0–3	0–45	0–70	SH
57	701–900	5–6	13–17	6–7	19–25	3–4	4–5	46–60	71–100	MH
4	901–1400	7–8	18–28	8–9	26–37	5–7	6–8	61–75	101–382	LH

*TH* total houses, *CA* carpet area, *R* rooms, *TRA* total regular appliances, *TLA* total lifestyle appliances, *TA* total appliances, *5Y* + *A* five-year-old appliances, *M* members, *QS_Equ* questionary survey equation, *Avg_kWh* average consumption, *HT* house type, *SD* standard deviation, *SM* small house, *MH* medium house, *LH* large house, *VLH* very large house

### Assumptions for the proposed SHOEC approach

The proposed knowledge-based decision-making system for energy bill evaluation and optimization is the first socio-inspired method for household electricity consumption and energy bill optimization using the SHOEC algorithm. The direct comparison between the Socio-technical Household Optimized Electricity Conservation (SHOEC) results and the existing models or techniques for the household electricity consumption and optimization study is challenging. This challenge is due to variations in the implications, proposed methodology, data-collection methods, data characteristics, selected variables, and the used validation indicators that can be applied differently in different works, which can vary the results of SHOEC. The performance of the SHOEC can be compared or verified, when the optimization model works on the same data sets, methodology, and implications. If compared, every optimization model has its potential and limitations. Thus, one algorithm cannot perform the best on all data sets and applications under all conditions^1–3,62,63^. The SHOEC results are explored to define the thumb rule to understand and validate household electricity consumption and optimization.

### Result verification using genetic algorithm

Tables 9 and 10 show the objective function used for different categories of houses and optimal solutions, compared with the origin consumption for validation. Thus, the proposed algorithm performs similarly to other comparison algorithms.

**Table 9:** Type A houses with linear objective functions and constraints using GA optimization.

House type	House usage	Objective function	Constraints	Conditions
TypeA_SH_H1	LEDH	Y(A)1 = 5R + 1A + 7 M	6R + 19A + 5 M ≤ 100	High
			3R + 15A + 3 M ≤ 70	Medium
			2R + 10A + 1 M ≤ 35	Low
TypeA_MH_H2	MEDH	Y(A)2 = 12R + 2A + 15 M	7R + 37A + 7 M ≤ 250	High
			4R + 20A + 4 M ≤ 160	Medium
			3R + 20A + 3 M ≤ 130	Low
TypeA_LH_H3	PEDH	Y(A)3 = 22R + 4A + 30 M	9R + 45A + 7 M ≤ 350	High
			7R + 35A + 4 M ≤ 250	Medium
			4R + 30A + 3 M ≤ 120	Low

*R* rooms, *A* appliances, *M* members, *SH* small house, *MH* medium house, *LH* large house, *LEDH* low energy demand house, *MEDH* medium energy demand house, *PEDH* peak energy demand house.

**Table 10:** Type B houses with linear objective functions and constraints using GA optimization.

House type	House usage	Objective function	Constraints	Conditions
TypeB_SH_H2_1	MEDH	Y(B)1 = 15R + 3A + 20 M	6R + 27A + 6 M < 250	High
			4R + 20A + 3 M < 170	Medium
			2R + 10A + 2 M < 115	Low
TypeB_MH_H3_1	PEDH	Y(B)2 = 18R + 5A + 20 M	7R + 35A + 6 M < 350	High
			4R + 25A + 4 M < 200	Medium
			3R + 20A + 3 M < 150	Low

*R* rooms, *A* appliances, *M* members, *SH* small house, *MH* medium house, *LH* large house, *LEDH* low energy demand house, *MEDH* medium energy demand house, *PEDH* peak energy demand house.

The result gives the authors a ground-level study to further establish the effectiveness of this metaheuristic by solving objective and real-world problems. Thus, GA is a Stochastic approach. Based on the dataset availability objective function, constraints are developed for different house sizes, and different dataset input-based results are calculated for energy consumption, which is almost similar to the socio-technical competitive behavioral approach which validates that the proposed method is working well^12,13^.

## Discussion and conclusions

### Discussion

A manual meter reading and billing systems of MSEDCL utility of domestic electricity consumers based on energy conservation and bill issues are addressed. The manual meter reading and billing process has two main ways to generate error bills: (i) human error at energy meter photo reading and punching of reading in the machine and (ii) machine error at energy meter and mobile or app^6,49^. Meanwhile, the utility company has its energy bill verification system before generating the energy bill. The existing energy bill verification system in the conventional grid system is generalized and threshold-based, applicable to all consumers. This can lead to inadvertently high or erroneous energy bills for consumers. To overcome this problem, consumers with more elevated consumption slabs can pay higher bills. Thus, a generalized threshold-based approach may only be effective and equitable for some consumers.

A Knowledge-based Decision-making Support system for energy bill Evaluation and Optimization (KDSEO) approach addresses energy bills and their evaluation issues. This system works on household electricity consumption understanding and achieving energy bill optimization through energy conservation, cost savings, and social impact aspects; for KDSEO, a peer-and self-group competitive behavior-based Socio-inspired Optimization (SO) method is used. SO is a new category of evolution optimization method and has yet to be attempted for household electricity consumption studies. The SO methodology proposes a Socio-technical Household-Optimized Electricity Conservation (SHOEC) algorithm. Thus, KDSEO aims to classify energy bills, categorize households, and use the SHOEC algorithm to optimize energy consumption with the EB assessment and optimization.

The socio-inspired optimization approach is employed to develop an energy bill optimization model. This work performs optimal house classification based on MC and QS datasets, with the conduction of self and peer-comparative energy consumption analysis. The input data comprises a Questionnaire survey (QS) and monthly data on household consumers' energy consumption (MC) in kilowatt-hours (kWh). The QS consists of different sections for collecting information, including general information, energy consumption details, home characteristics, socio-economic factors, types of appliance, feedback, and awareness. The QS data has been collected online via Google Forms and in hardcopy through offline modes. The MC data is gathered from household consumers and the utility, encompassing individual household monthly historical electricity consumption in kWh, energy meter status, and Go-Green activation status. Using this data, several data preprocessing techniques, Feature Engineering (FE), clustering, classification, and prediction modeling are applied, and the output is integrated into the socio-inspired optimization model^22^.

In the FE technique, new attributes are derived. Based on the QS dataset, five significant details are generated: the summation of socio-demographic parameters, the total count of regular appliances, the total count of lifestyle appliances, the number of five-years-old appliances, and the summation of significant QS attributes (QsSum). The QsSum attribute is created by adding up the values of the above four attributes. QsSum is then utilized to define the house size for individual houses. Similarly, in the MC dataset, six significant details are considered: MC data, maximum energy consumption, minimum energy consumption, standard deviation, summation of monthly energy consumption, and average monthly energy consumption (Avg_kWh). In addition, the houses are classified based on the summation of significant attributes of QS of individual homes (QsSum) and the average energy consumption (Avg_kWh) detail from MC. The houses are Small Houses (SH), Medium Houses (MH), Large Houses (LH), and Very Large Houses (VLH). Both QsSum and Avg_kWh types of houses are explored and categorized for energy bill evaluation and optimization. They further predict monthly energy consumption with the help of a prediction model called SMSDAR.

The SMSDAR prediction model contains a Support Vector Machine (S), Multi-layer perceptron (M), Stochastic Gradient Descent (S), Decision tree (D), Adaptive boosting (A), and Random Forest (R). In addition, the energy-saving tips and energy conservation behavioral-based approach points are proposed after interacting with household consumers during field visits. There needs to be more consumer awareness of energy conservation and waste^58,59,64,65^. Household consumers can take some doable initiatives to improve energy efficiency and lower the electricity bill. First, to avoid standby consumption, it is suggested that home appliances should be turned off from the main switchboard or left unplugged while not in use. Electronic appliances, including TVs, set-top boxes, air conditioners, desktop and laptop computers, Wi-Fi routers, laundry machines and video game consoles are controlled by a remote and forgotten on the switchboard. Energy savings can also be achieved by efficient refrigerator utilization, which includes restricting stored products, adjusting the changing temperature, and defrosting the freezer once a month. Another tactic to reduce the frequency of use is to run appliances, such as washing machines, at full load. Give preference to using natural resources, such as wind and sunlight, for ventilation, lighting, and dish and laundry drying. Installing rooftop solar panels and net metering can reduce energy bills with initial costs. Furthermore, it is advised to install Earth Leakage Circuit Boards (ELCB) to identify and stop leakage currents from causing excessive billing. Using these techniques, households can optimize energy consumption and can save significant amount of electricity.

### Advantages

The paper's main contribution is to propose a methodology for developing a knowledge-based decision-making system to verify the household consumer's monthly energy bill using the Knowledge-based Decision-making System for energy bill Evaluation and Optimization (KDSEO) system. This work contributed to the existing body of knowledge that coined the socio-inspired approach under cultural or social optimization named socio-technical competitive behavior, as shown in Fig. 1. Further, the proposed KDSEO system aims to generate an energy bill report, providing information to end consumers to evaluate bills and make informed decisions. The primary outcome focuses on energy and cost optimization for consumers, while the secondary outcome encourages active consumer participation in the billing process and contributes to reducing carbon emissions^1–4^. In addition to this, the occupation offers several remarkable benefits. First, it obtains optimal household classification, using an average monthly energy consumption and Questionary survey data.

Furthermore, the energy bill report of Knowledge-based Decision-making System for energy bill Evaluation and Optimization system and proposed thumb rule give the end household customers more power by letting them examine their monthly energy bills, making it easier to identify typical energy-use patterns and instances of abnormally high consumption. The energy bill report provides three household categories based on their usage: excellent, perfect, and poor. This can create a competitive environment for household consumers to compare, understand, change, and improve energy conservation.

### Limitations

The restricted geographical scope of this research bounds its usefulness to a specific region or area. The implementation of the study in a limited geographic area raises the possibility of the conclusions and understandings gained from it are context-specific and challenging to apply to other contexts. Additionally, the study uses a manual meter reading and billing system, which is a meter reading procedure relying on a traditional grid system. Moreover, other consumer categories like industries or customers are not involved in the research work; instead, they concentrate exclusively on the household consumer category. This focused method makes it possible to examine home electricity consumption studies, but it may also limit the completeness and generalizability of the study to a broader range of users. Conclusively, the geographic restriction, dependence on manual grid systems, and exclusive emphasis on residential consumers, delineate the particular criteria and limits, that direct the conduct of this research, impacting the scope and possible implications of its findings^1–4^.

### Conclusions

This paper proposes a knowledge-based decision system to evaluate monthly energy consumption, to avoid overloading energy consumption billing. This approach will inform the consumers about the verification of bills and suggest possibilities for energy conservation. Here, a socially driven approach is used for energy bill assessment. The knowledge-based system includes data collection, pre-processing, feature engineering, home classification, and monthly energy forecasting. In addition, the system classifies the energy bill of the consumer as normal or high. The consumer can compare their energy usage for the same home size as the system classifies the consumer as excellent, perfect, and poor usage. The 225 households have been considered in this study, and the final 139 have been used to develop the decision-making system. Our system recognizes that 31% of bills are high and 69% bills are normal. In addition, the performance of this analysis is verified using linear programming and genetic algorithm-optimized techniques. This helped to provide optimized energy consumption for different household categories based on socio-demographic constraints as another approach. The results of the adapted methods are almost identical to those of peer and self-group behavioral techniques. The result shows that the proposed system has performed better regarding energy conservation and cost-saving solutions. The proposed study allows consumers to find the optimum energy consumption solution and effectively reduce the bills of the individual households.

### Future scope

To enhance the robustness and applicability of this research, future work can include using the proposed methodology in various regions, utilizing smart-meter datasets with more comprehensive consumption data histories. The different consumer categories, like commercial and industrial, can be the focus and can improve our understanding of the dynamics of energy usage. It would also improve the study by considering different variables, namely behavioral consumption patterns, types of days (workdays, weekends, etc.), seasonal variations (summer, winter), weather parameters (temperature, wind speed, humidity, etc.), and appliance-based consumption data. Another area of research applies different Machine Learning and Deep Learning techniques, including supervised, unsupervised, and reinforcement, for optimal household classification. Additionally, there is scope to examine the impact of the calendar and seasonal variability attributes on individual appliances' usage patterns and to analyze the effect of these attributes on the prediction model. This work has overall potential to provide broadly applicable findings in the field of household electricity consumption analysis, by expanding the geographical and demographic scope, adding other factors, and utilizing cutting-edge Machine Learning techniques.

### Supplementary Information

Supplementary Information.

## Data Availability

The datasets generated and/or analyzed during the current study are not publicly available due to the data that has been used being confidential, and the study is yet to be completed but is available from the corresponding author at reasonable request.
