# Supplementary material for: A peer-and self-group competitive behavior-based socio-inspired approach for household electricity conservation
Source: Sci Rep. 2024 Jul 27;14:17245. doi: 10.1038/s41598-024-56926-1 (PMC11282066; doi:10.1038/s41598-024-56926-1)
Supplement: Supplementary file 1 — Supplementary Information. [file 41598_2024_56926_MOESM1_ESM.docx]

**Survey on Household Electricity Consumption for PhD Work**

***(Consent Cum Survey Form)***

Dear Participant,

I am conducting a survey on "Energy Optimization at Household" as a part of academic research (PhD) work. I request you to give your honest, candid response and cooperation. Based on your response, I can give electricity bill related improvements to you and energy service provider company like MSEDCL/MSEB/Mahavitaran company. The data collected will be used exclusively for academic research purposes only, without disclosing your identity. Based on random sampling collecting around 500 household’s survey located in Maharashtra state. This basic survey will take around 15 minutes to complete, and if any query I will connect to you or some time. I thank you for participating in this study.

Sincere Regards,

Gaikwad Sachin Ramnath,

Junior Research Fellow (JRF),

Dept. of Electronics and Telecomm. Engg.

Symbiosis Institute of Technology (SIT),

Symbiosis International Deemed University (SIDU), Pune- 412115.

Mobile No.- +91-9604088993/9309171936

Email ID.- [sachin.r.gaikwad@outlook.com](mailto:sachin.r.gaikwad@outlook.com)/sachin.gaikwad.phd2019@sitpune.edu.in

- **Name of Participant (Optional):** ……………………………………………………………
- **Household Address (Full):** …………………………………………………….....................
- **Email Address:** ………………………………………………………………………………
- **Mobile Number:**……………………………………………………………………………..


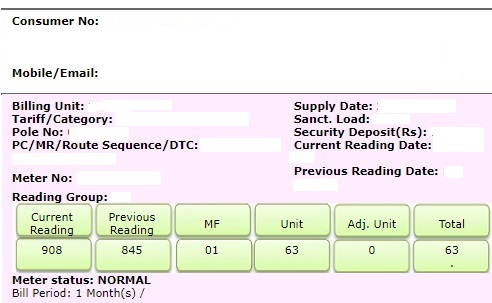


**Supplementary Figure 1. Sample electricity bill.**

- **Consumer Number** (Refer the electricity bill, 12-digits): …………………………..............
- **Billing Unit Number** (Refer the electricity bill, 4-digits): ……………………….................
- **Your energy meter status** (Below the current and previous reading): Normal/ Average/ Faulty/Other
- **Type of household:** Owner/ Tenant or Renter
- **How long do you live at this house:** (since date:……./...../...........)
- **Your home carpet area in square feet (Sq.ft.)**: ………………………………………….
- **Number of rooms (Including hall, kitchen, and toile-bathroom):** …………………….
- **Number of balconies to your home:** ……………………………………………………….
- **Number of windows (Except toilet-bathroom):** ………………………………………....
- **What AC temperature you set at your house:** ……………°C (If you use AC in home)
- **Total family members at your home:** …………………………………………………….
- **Total monthly income of your home:**

1. Less than Rs. 50,000/-
2. Between Rs. 50,000/- to 1 Lack
3. More than 1 Lack
4. Would not like to disclose

- **Highest level of education at your house:** Below 10^th^/10^th^/12^th^/Diploma/Graduation/Post graduation/ Phd / Other
- **Write last twelve months units consumed from latest electricity bill**: .……../……...../…….../…..…./…..…./…..…./…..…../…..…./….…./…..…./…….../….…../

**(Example: 17/18/20/17/13/22/66/125/118/65/46/55)**


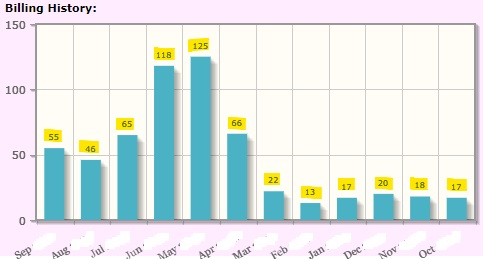


**Supplementary Figure 2. Consumption history in units (kWh)**

- **Power-supply connection to your home:**

1. Maharashtra State Electricity Board/MSEB/MSEDCL
2. Roof-top solar panel
3. Both 1 & 2
4. Other

- **Type of power outage/power cut/ power failure at your home:**

1. Frequent and unpredictable 2. Infrequent and unpredictable

3. Regular and unpredictable 4. Occasional 5. Can’t say 6. No outages

- **Voltage fluctuation at your home:** Yes / No / Can’t say
- **Your home location at your building.** (If not in building then select 4/5 option)

1. Ground floor
2. Top floor
3. Other floor
4. I am living at independent house
5. I am living at row house

- **Facing of your home door direction:** East / West / North / South
- **How is the ventilation and sun-lighting at your home?**

Excellent / Satisfactory/ Not satisfactory

- **Do you turn off your major household appliances from switch board when they are not in use?** (e.g. - AC, TV, setup-box, laptop, sound system, wireless router, etc.)

Always/ Sometimes/ Never

- **Do you turn off all the lights when you are leaving a house:** Always / Sometimes / Never
- **Does your house have tree shade?** Yes / No
- **Your house is neighboring to any water bodies?** (e.g. rivers, lakes, etc.) Yes /No / Can’t say
- **Do you use ELCB (Earth Leakage Circuit Breaker) at your home:** Yes / No / Can’t say


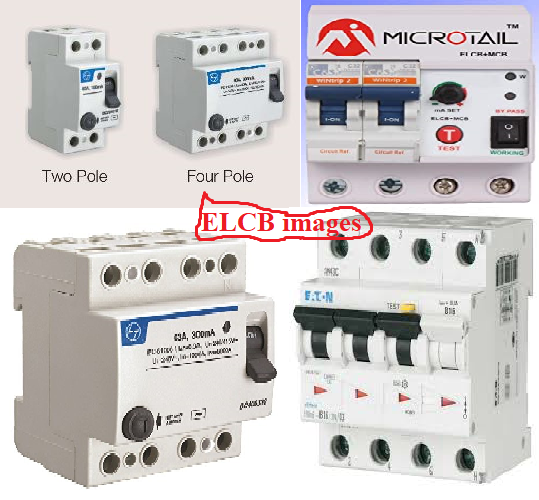
**Supplementary Figure 3. samples of Earth Leakage Circuit Breaker**

**
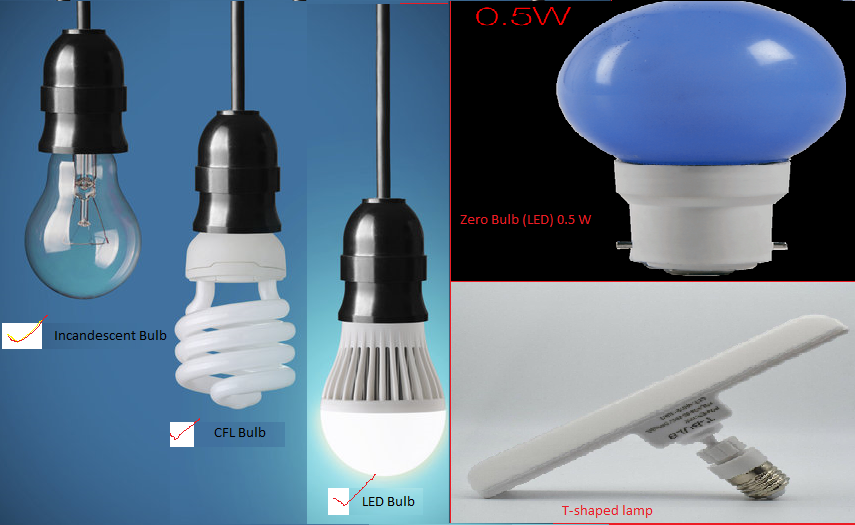
**

**Supplementary Figure 4. Types of bulbs**

- **Select the equipment’s/ appliances you use at your home:**

| **Appliance Name** | **No.** | **Appliance Name** | **No.** |
| --- | --- | --- | --- |
| Television (TV) |  | Incandescent Bulb |  |
| Refrigerator |  | Fluorescent Tube Light |  |
| Fans |  | Inverter (Power Bank Ah) |  |
| Air Cooler |  | Water-Purifier |  |
| Air Conditioner (AC) |  | Water Motor Pump (HP) |  |
| Washing Machine |  | Electric Iron/ Press |  |
| Mobiles |  | Electronics (Video) Games |  |
| Mobile Power Bank |  | Electric Water Heater |  |
| Laptops |  | Electric Geyser |  |
| Desktop Computer |  | Microwave-Oven |  |
| Wifi Router |  | Induction Cooktop |  |
| LED Tube Light |  | Mixer or Blender (cooking) |  |
| LED Bulb |  | Others, please specify bellow: |  |
| LED Bulb (T-shape) |  |  |  |
| CFL Bulb |  |  |  |
| Zero bulb |  |  |  |

- **Select the equipment’s/appliances of more than 5 years old at your home**

(Note: Please do the tick mark (√) in box)

| **Appliance Name** | **Mark (√)** | **Appliance Name** | **Mark**  **(√)** |
| --- | --- | --- | --- |
| Television (TV) |  | Electric Geyser |  |
| Refrigerator |  | Electric Iron/Press |  |
| Fans |  | Electric Water Heater |  |
| Air Cooler |  | Others, please specify below |  |
| Air Conditioner (AC) |  |  |  |
| Washing Machine |  |  |  |

- **Select below used star-labeled appliances at your home:**

| **Appliance Name** | **Can’t Say** | **Mark**  **(√)** | **Appliance Name** | **Can’t Say** | **Mark**  **(√)** |
| --- | --- | --- | --- | --- | --- |
| Television (TV) |  |  | Washing Machine |  |  |
| Refrigerator |  |  | Air Conditioner (AC) |  |  |
| LED Lamp |  |  | Electric Geyser |  |  |
| Florescent Tube |  |  | Others, please specify |  |  |
| Ceiling Fan |  |  |  |  |  |


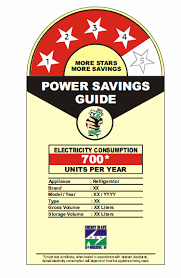


**Supplementary Figure 5. Sample of star label on appliance**

**Any suggestions/problems from consumer side:…………………............................................** ……………………...................................................................................................................
